# Membrane protein structures without crystals, by single particle electron cryomicroscopy

**DOI:** 10.1016/j.sbi.2015.07.009

**Published:** 2015-08

**Authors:** Kutti R Vinothkumar

**Affiliations:** Medical Research Council Laboratory of Molecular Biology, Francis Crick Avenue, Cambridge CB2 0QH, United Kingdom

## Abstract

It is an exciting period in membrane protein structural biology with a number of medically important protein structures determined at a rapid pace. However, two major hurdles still remain in the structural biology of membrane proteins. One is the inability to obtain large amounts of protein for crystallization and the other is the failure to get well-diffracting crystals. With single particle electron cryomicroscopy, both these problems can be overcome and high-resolution structures of membrane proteins and other labile protein complexes can be obtained with very little protein and without the need for crystals. In this review, I highlight recent advances in electron microscopy, detectors and software, which have allowed determination of medium to high-resolution structures of membrane proteins and complexes that have been difficult to study by other structural biological techniques.

## Highlights

• Electron microscopy of membrane proteins as single particles.
• Membrane protein structures without crystals.
• Direct electron detectors have high signal to noise.
• Medium to high-resolution structures of molecules between 0.13 and 2 MDa.
• Sub-tomogram averaging to study membrane proteins *in situ*.

**Current Opinion in Structural Biology** 2015, **33**:103–114

This review comes from a themed issue on **Membranes**

Edited by **Bruno Miroux** and **Eva Pebay Peyroula**

For a complete overview see the Issue and the Editorial

Available online 01st October 2015

**http://dx.doi.org/10.1016/j.sbi.2015.07.009**

0959-440/© 2015 The Author. Published by Elsevier Ltd. This is an open access article under the CC BY license (http://creativecommons.org/licenses/by/4.0/).

## Introduction

Membranes and membrane proteins have always fascinated electron microscopists because they were prominent features in the early electron micrographs of the cells. In the early days, electron micrographs were used to infer the organization of the lipid and protein components and for postulation of the fluid-mosaic model of membrane structure [1]. Specialized membranes with abundant proteins were also identified by electron microscopy, often with the constituent proteins arranged in crystalline arrays [2, 3]. This paved way for a separate discipline within electron microscopy, which was later called electron crystallography. The pioneering work of Henderson and Unwin on glucose embedded two-dimensional (2D) crystals of bacteriorhodopsin (also called purple membrane) revealed the first architecture of an integral membrane protein [4, 5]. Subsequent development of the rapid freezing of specimens in thin aqueous films gave rise to the field of electron cryomicroscopy (cryoEM) [6••, 7]. In combination with the introduction of field emission guns and better vacuums in the electron microscopes, this led to a great expansion of cryoEM, including an atomic model of bacteriorhodopsin [8, 9]. It was realized at the same time that membrane proteins in presence of lipids can also form helical tubes, which by helical reconstruction can also provide high-resolution maps as shown for the nicotinic acetylcholine receptor (AChR) [10]. Thus, in the 90s when obtaining well-diffracting three-dimensional (3D) crystals of membrane proteins for X-ray crystallography was still difficult, electron microscopy of membrane proteins in the form of 2D crystals or helical tubes was seen as an alternative approach to obtain structures of membrane proteins. The added advantages of the native environment and the ability to study conformational changes as shown for bacteriorhodopsin and AChR [11, 12] prompted many researchers to pursue electron crystallography. However, it soon became clear that obtaining well-ordered 2D crystals or tubes were just as difficult as obtaining well-ordered 3D crystals. Although many membrane proteins formed 2D crystals immediately, often they were poorly ordered resulting in medium to low-resolution maps, with only a handful of structures determined to high-resolution [13]. With the advent of genome sequence determination and identification of homologues of many important membrane proteins, there has been a rapid increase in structures of membrane proteins mainly by 3D crystallization and X-ray crystallography, superseding electron crystallography [14].

Obtaining sufficient membrane protein and well-diffracting crystals still remains an obstacle, and having worked on membrane proteins for a while, I have always wondered if it is possible to completely get away from crystals. Two techniques that have the potential to provide high-resolution structures of membrane proteins (or any macromolecule) without crystals include nuclear magnetic resonance spectroscopy (NMR) and single particle cryoEM. Of these, structural determination of membrane proteins by NMR requires a large amount of labeled protein and stability in a given detergent for at least few days during measurement [15]. For many membrane proteins, in particular those from eukaryotes this may not be feasible. In single particle cryoEM, membrane proteins in solution are rapidly frozen and imaged with electrons as single molecules in a thin film of buffer. By averaging a large number of particles, high-resolution structures can then be obtained with very little protein. This technique has an added advantage that distinct structural states in a solution can be computationally separated and multiple structures of the same protein determined.

## Single particle EM

**Figure 1:**
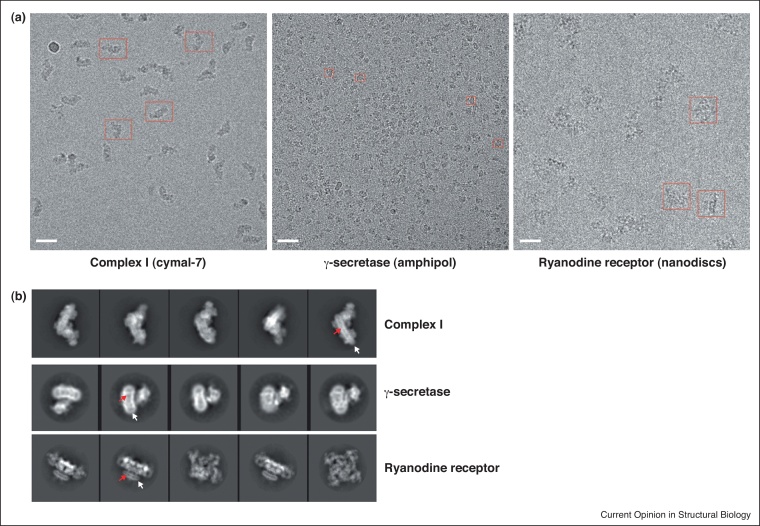
Membrane protein imaging as single particles by electron cryomicroscopy. **(a)** Selected areas of micrographs of membrane proteins observed in detergent (Complex I), amphipol (γ-secretase) and nanodisc (ryanodine receptor). The images of Complex I and RyR were taken with the FEI Falcon II detector and the γ-secretase was imaged with the Gatan K2 Summit detector. Scale bar is 400 nm. In the case of RyR receptor, fluorinated octyl maltoside was added to the protein solution just before freezing to get better distribution of the receptor [38^•^]. The high contrast seen in these images is due to the use of relatively high dose and sufficient defocus. For example, the Complex I images were captured for 4 s (72 frames) at a total dose of ∼68 electrons/Å^2^. This high dose image was only used for particle picking and contrast transfer function estimation. For subsequent refinements the last 40 frames were discarded [58^•^]. **(b)** Reference-free 2D class averages of Complex I, γ-secretase and ryanodine receptor. The panel shows a selection of 2D class averages of different membrane proteins revealing the prominent detergent/lipid belt around the protein (visible as a band around the membrane part and marked with white arrow), transmembrane (TM) helices in the membrane domain (marked with red arrow) and the quaternary structure. In the case of Complex I, the location of some of the supernumerary subunits can be seen in the class averages. The box sizes in the 2D class averages are 280, 90 and 384 pixels for Complex I (1.72 Å/pixel), γ-secretase (2.8 Å/pixel) and ryanodine receptor (1.45 Å/pixel) respectively.

Electron micrographs of single protein molecules contain projections of the underlying structure of the specimen and due to the limited electron dose required to minimize radiation damage have a poor signal to noise ratio. Averaging many projections increases the signal and from these it is possible to generate a three-dimensional 3D reconstruction (see Figure 1 for examples of electron micrographs and class averages). The basic computational principle in single particle cryoEM involves accurate determination of five parameters: namely the three Euler angles and the *x*,*y* translations. A simple rule suggests that the more accurate the orientation of the particles, the higher the resolution. Often, this depends on various parameters including the signal to noise ratio, orientation distribution of the particles that is if all possible views of the molecule are observed, and structural heterogeneity. One or all these parameters affect the achievable resolution. From theoretical estimate, Henderson has calculated that if perfect images of macromolecules can be obtained from an electron microscope then high-resolution structures can be reconstructed for a wide range of macromolecules by averaging only few thousands of asymmetric units [16••, 17•]. He pointed out that radiation damage, beam-induced movement and signal to noise ratio in images were the major limiting experimental factors, which when solved would allow to obtain perfect images and higher resolutions as predicted from theory.

The advent of microscopes with coherent beams, better vacuums and optics allowed the first atomic resolution maps to be obtained by single particle reconstruction of highly symmetrical macromolecules with images taken on film or a charged-coupled device (CCD) [reviewed in 18]. These maps showed that it was indeed possible to obtain high-resolution structures without crystals. In the last couple of years, with the introduction of direct electron detectors, it is now possible to pursue structural studies of less symmetrical macromolecules that were otherwise difficult to determine. The advantages of the direct electron detectors can be summarized in the following points: 1) higher detective quantum efficiency (DQE) or simply higher signal to noise ratio [19]; 2) the possibility to look and evaluate the quality of the specimen immediately (unlike films, which had to be developed and checked by optical diffraction) and collect more data in a given period of time; 3) as they operate in rolling shutter mode, the ability to dose-fractionate the exposure allows one to computationally correct for drift and beam-induced motion and choose selected frames and electron dose that gives the best reconstructions [20, 21, 22, 23, 24]. There are several reviews that chart the development of cryoEM from its early history to the present day advances [25, 26, 27, 28, 29, 30, 31••], which I highly recommend to readers but this review will mainly focus on single particle studies of membrane proteins.

In the early days, electron microscopy of membrane proteins as single particles was largely limited to visualizing them with heavy metal staining. This was based on the general thought that due to the presence of detergents it would be difficult to get sufficient contrast in ice. However, studies on the mitochondrial Complex I [32], hexameric P-type H^+^-ATPase from *Neurospora crassa* [33] and the bovine F_1_F_o_ ATPase [34] showed that single particle imaging of membrane proteins in ice was possible. Despite their limited resolution, the calculated maps were informative and reflected the true structure of protein (not the envelope from the heavy metal stain). The structure of bovine ATPase in particular remains a major landmark as it provided an envelope for an intact multi-subunit membrane protein complex on which crystal structures of several known subunits could be docked [35].

## Seeing is believing — biochemistry is vital

A great advantage of EM is the ability to directly visualize the protein of interest and tune the biochemistry to optimize the specimen for any given investigation. The homogeneity of membrane proteins is generally judged based on gels and gel-filtration profiles, occasionally complemented with functional assays. However even a single band on a gel or a symmetrical gel filtration profile may not reflect the true nature of membrane protein in solution. When observing such solutions by cryoEM, one may sometimes be surprised to see a wide variety of features including lipid-detergent mixtures either as vesicles or small membranes, denatured protein or dissociated subunits. If the membrane protein of interest is spherical then it can be difficult to judge the quality of biochemistry amid such background. With multi-subunit membrane protein complexes, the dissociation of subunits can sometimes occur during freezing of protein solution on grids and some understanding behind the process of blotting and freezing is thus beneficial [6^••^]. In the initial stages of studying a membrane protein by single particle cryoEM, it is often useful to image them with a combination of high dose (when using direct detectors) and high defocus, so that they are clearly visible and some features of the protein can be recognized. This is particularly useful for smaller membrane proteins. Such high-contrast low noise images provide a wealth of information about the purity and integrity of the sample but also prevent picking features that could be just low-contrast random noise [36].

Just as in crystallization, the choice of detergent or its substitutes and the final detergent concentration plays a critical role in cryoEM. Too high a concentration of detergent adds to the background. When possible it is ideal to use the protein directly off the column without a concentration step. Membrane proteins have been observed as single particles in detergent micelles, amphipol, liposomes and nanodisc (Table 1 and Figure 1a). To date, except for liposomes, all other media have produced medium to high-resolution structures so at present it is not possible to generalize about whether a particular detergent or its substitute is best suited for cryoEM or is essential to obtain high-resolution. The recent structures of ryanodine receptor (RyR) in detergent [37^•^], nanodiscs [38^•^] or mixed lipid-detergent micelles [39^•^] reveal similar structures and highlight the fact that the specimen preparation is likely to play a more crucial role (e.g. thickness of ice) rather than the choice of detergent or its substitute. For a given membrane protein, it is important to find the right mixture of detergent/lipid that keeps in its functional and native state rather than following a particular protocol that has been found to be successful for other proteins.

Reference free 2D class averaging, where particles with similar views are averaged to produce projections of the structure that are less noisy and can further provide assessment of the quality of the protein prep. This requires only a few thousand particles and perhaps 50–100 images. In Figure 1b, examples of 2D class averages of different membrane proteins are shown. They clearly reveal distinct views, internal details such as the transmembrane (TM) helices and the detergent/lipid belt around the membrane domain. The quaternary structure, integrity and conformational heterogeneity of the proteins become prominent in such class averages (e.g. the tetrameric arrangement of RyR in Figure 1b). Analysis from specimen preparation to imaging and 2D classification can be done in a relatively short period and provides a good feedback about biochemistry before large-scale data collection and reconstruction is attempted. Such screening of membrane proteins and their complexes, either by negative staining or by cryoEM, has been instrumental in identifying the right constructs, leading to better crystals and structures and to study underlying structural heterogeneity in solution [40, 41, 42].

## From blobs to high-resolution structures

In the early days of cryoEM of membrane proteins, a large number of reconstructions have been published but they showed relatively featureless blobs. There was also the separate problem that reconstructions of the same protein often yielded different results and interpretation. Further, the propensity to average and refine noise resulted in claims of resolution that could not be justified by the maps [43, 44]. Therefore, there was general skepticism about whether reliable, high-resolution maps could be obtained by single particle cryoEM. The maps of the ryanodine receptor [45, 46] (the membrane domain in these RyR maps were of lower resolution) and the A type-ATPase from *Thermus thermophilus* reconstructed from images taken on a CCD or film were the first examples to show that sub-nanometer resolution of membrane proteins by cryoEM was possible [47^•^]. Multiple structures of membrane proteins (the translocon, YidC) in complex with ribosomes have been described but the resolutions of the membrane protein part in many of these reconstructions are generally lower than ribosomes and often the TM helices are not clearly resolved [48, 49, 50, 51, 52, 53, 54]. Nevertheless, they have provided important biological insights into the mechanism of protein translocation.

**Figure 2:**
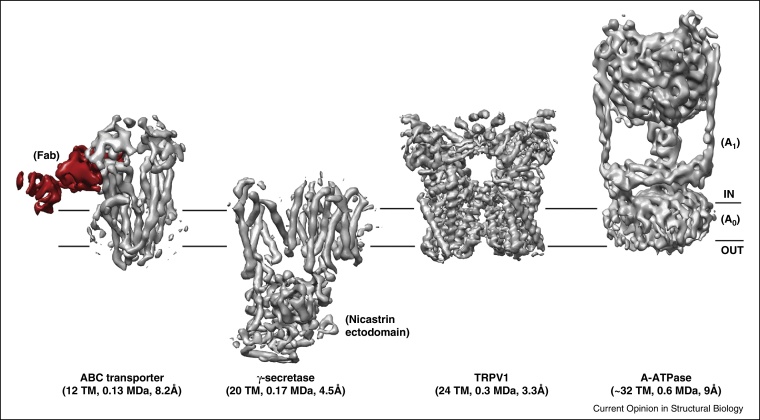
A gallery of membrane protein structures determined by single particle cryoEM. The maps have been selected to show a range of molecular weight, complexity of the proteins and resolution. The heterodimeric ATP-binding cassette (ABC) transporter from *Thermus thermophilus* has 12 TM helices with an extended cytoplasmic ATP binding domain and has a molecular mass of ∼130 kDa. Such a small protein by itself can be visualized and a ∼10 Å map has been obtained [59^•^]. A complex of this ABC transporter with an Fab antibody fragment (50 kDa, shown in red) that recognizes one of the subunit aids in more accurate orientation resulting in an 8 Å map that clearly resolves the TM helices. γ-Secretase is an intramembrane aspartyl protease that cleaves a wide range of single pass TM substrates but widely known for cleaving amyloid β-peptide. Four different subunits (PS, PEN-2, APH-1 and Nicastrin) are essential for assembly and functionality of the enzyme. The protein mass of γ-secretase is ∼170 kDa and the presence of the large ectodomain of Nicastrin greatly assists the alignment of the particles and a map at overall resolution of 4.5 Å was obtained. The ectodomain is at higher resolution and much of the path of the polypeptide could be traced [56^•^]. The TM domain of γ-secretase is at lower resolution but sufficient to reveal the arrangement of TM helices, providing insights into how the substrate might approach the interior of the enzyme. The transient receptor potential V1 (TRPV1) ion channel is a receptor for capsaicin and belongs to the family of ion channels that are involved in sensing and transducing temperature [55^••^]. The structure of TRPV1 at 3.3 Å is one of the highest resolution maps of a membrane protein determined by single particle cryoEM. The map shows the classical fold of the voltage-gated ion channels with 6TM helices per monomer in a tetrameric arrangement. Such a high-resolution map allowed de novo model building of almost the entire polypeptide chain [55^••^]. Multiple structures of TRPV1 in complex with inhibitors have been obtained providing insights into the possible gating mechanism and illustrate the power of cryoEM [95^•^]. The H^+^-driven A-type ATPase from *Thermus thermophilus* is a moderate sized membrane protein and the reconstruction was performed with images captured on film. Though of lower resolution (∼9 Å), the structure shows the global architecture of the A type-ATPases with the A_1_ catalytic domain, the A_o_ membrane domain, the central and the two peripheral stalks [47^•^]. The F and V-type ATPases have equivalent F_1_/V_1_ and F_o_/V_o_ domains that perform catalysis and ion translocation [60•, 64•]. The horizontal helices in the A_o_ membrane domain that we now know to exist from higher resolution maps of the F and V-type ATPases [60•, 64•] can now be correlated in this low-resolution map.

**Figure 3:**
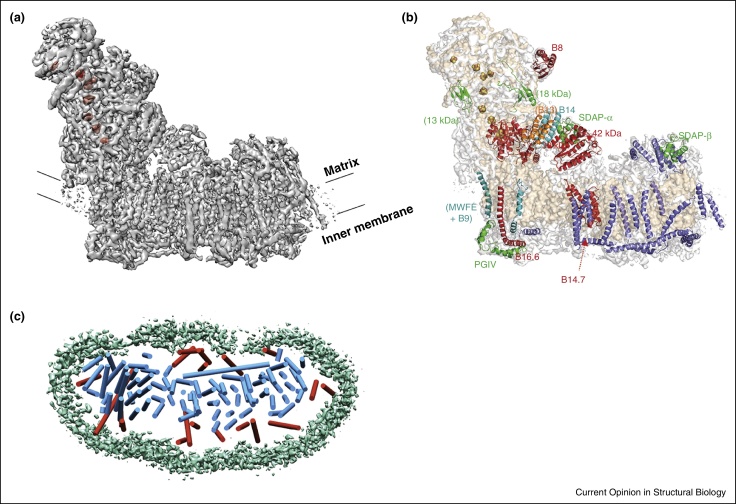
Architecture of mammalian respiratory Complex I. In the inner membrane of mitochondria (and the inner membrane of prokaryotes), the first step in electron transfer is the oxidation of NADH by a multi subunit complex called NADH:Ubiqunione oxidoreductase or commonly called Complex I. The electrons from NADH are transferred to ubiquinone through a series of iron-sulfur (FES) clusters and this transfer is coupled to proton translocation across the membrane. The core subunits as defined in prokaryotes harbor all the catalytic subunits for electron transfer and proton translocation [96]. In higher eukaryotes, the core subunits are augmented with varying numbers of accessory subunits that are involved in assembly and regulation and are called supernumerary subunits. The bovine Complex I is a biochemically well studied enzyme with a molecular mass of ∼1 MDa and is composed of >40 subunits [58^•^]. **(a)** A reconstruction of bovine Complex I at ∼5 Å was obtained by cryoEM imaging of the enzyme in detergent micelles. Two maps are overlaid to show two distinct feature of the enzyme. The eight FES clusters (shown in red) are visible as the highest peaks in the map and the density for protein (gray) at intermediate threshold shows a number of TM helices spanning across the membrane domain. For clarity, the detergent/lipid belt that is visible at lower threshold is not shown but the black bars mark the boundary defined by them. **(b)** Assignment of subunits to Complex I. The map of Complex I has a nominal resolution of ∼5 Å and some of the core regions in particular TM helices are better resolved and start revealing the bulky side chains. The 14 core of subunits of bovine Complex I share high homology with the prokaryotes. Using the atomic model of the *Thermus thermophilus* enzyme, the backbone and well resolved regions were built [96]. For the assignment of supernumerary accessory subunits, a combination of biochemical and genetic information, secondary structure prediction and subunits with known structures were employed. The identities of 14 supernumerary subunits have been assigned. These include subunits that are membrane embedded as well as soluble proteins. The assigned subunits are colored in red and green and labeled with the same color. The identities of the subunits whose names are labeled with brackets are less certain. Structural elements including several of the supernumerary membrane subunits with a single TM helix have not been assigned (blue) in the current map. Figure reproduced with permission from [58^•^]. **(c)** A view from matrix of mitochondria showing the arrangement of the TM helices in the membrane domain. The seven core membrane subunit TM helices are shown in blue and the TM helices of supernumerary subunits in red. In total, bovine Complex I has 78 TM helices. The curved nature of the membrane domain and the long horizontal helix are clearly visible. The detergent/lipid belt observed in the cryoEM map is colored in green.

With direct electron detectors, it is now possible to routinely visualize membrane proteins and obtain medium to high-resolution maps in a relatively short period (Table 1 and Figure 2, Figure 3). Some of the recent structures of membrane proteins determined by single particle cryoEM include transporters, enzymes, ion channels and multi-subunit membrane protein complexes [37•, 38•, 39•, 55••, 56•, 57•, 58•, 59•, 60•, 61•, 62•, 63•, 64•, 65]. The molecular mass of these proteins varies between 0.13 and 2.2 MDa. They contain 12–78 TM helices and many of them lack any symmetry making these structures truly ground breaking. The structures of RyR and ribosome in complex with Sec61 clearly highlight the advantage of the direct detectors over film or CCD with an increase in the resolution of the reconstructions despite using fewer particles [37•, 38•, 39•, 66].

Many membrane proteins by nature are dynamic and can exhibit multiple conformations in solution. For crystallization, such structural heterogeneity has been overcome by stabilizing a particular conformation using mutagenesis, antibodies or inhibitors. In cryoEM, such structural heterogeneity can be computationally sorted into distinct classes using recently introduced maximum likelihood based 3D classification, which results in maps that are less noisy and far superior [67, 68]. Many of the membrane protein structures listed in Table 1 have greatly benefitted from such 3D classification and I foresee that this is one area in image processing where future improvements in software will help immensely in obtaining higher-resolution maps of membrane proteins in distinct conformations with less particles.

One of the key areas in cryoEM where there has been lot of ambiguity is the correctness and the actual resolution of the maps [69]. In the past few years, several tests have been introduced to validate cryoEM maps. Of these, the tilt-pair validation test is a very powerful technique, where two images are taken from the same area at different tilt angles; this then allows the orientation of a given particle to be checked against a map that is thought to represent the protein of interest. The handedness of the map can also be determined with this technique [17•, 70]. CryoEM images are noisy and invariably noise builds up during the 3D refinement. High-resolution noise substitution or randomization of phases beyond certain resolution is an elegant way to check over-fitting of the data [71]. Recently introduced ‘gold-standard refinement’ aims at preventing such over-fitting already during refinement [72, 73]. As the data quality has improved, the maps have become better and it has become now standard to expect certain structural features to be seen with a given claim of resolution. For instance, maps at resolutions better than 10 Å should clearly show the separation of α-helices. Resolving β-strands at ∼4.5 Å and the appearance of side chain densities of larger amino acids at resolutions beyond 4 Å are the next landmarks in judging the resolution of the maps. It is also becoming clear that the resolution often varies across the macromolecule with the core regions of the protein generally show higher resolution than the peripheral regions of the molecule. It is now common in cryoEM publications to show a figure of the map depicting the local resolution [74]. The gallery of membrane protein structures in Figure 2, Figure 3 are in the resolution range of 3.3–9 Å and illustrate the structural features that one would expect for each of these resolutions. As the resolutions have started to reach 3 Å or above, bound ions and ligands are now being built confidently. Such model building and refinement on cryoEM maps have benefitted from modifications of existing crystallographic tools [75, 76, 77, 78].

For a biochemically well-characterized reasonably sized membrane protein, with the present technology it is now routinely possible to get maps below 10 Å with very little effort. Depending on size, symmetry and sample heterogeneity, achieving higher resolutions may take a substantial amount of time. The overall B-factor of the map, which reflects the quality of the images and orientation accuracy, is a useful indicator on how well the refinement is progressing and if the imaging conditions are optimal. In particular, it provides an estimate to the additional number of particles required to reach a particular resolution [17^•^]. Thus, it is advisable to set intermediate goals and it may be useful to improve the biochemistry, specimen preparation and imaging rather than averaging millions of particles to find that the resolution of the reconstruction increases only marginally. Even at medium resolutions (such as 5–7 Å) biological insights can be inferred. Such examples from recent structures include the description of the arrangement of TM helices and the position of the ectodomain in γ-secretase, the identification of horizontal TM helices in ATPase's and the assignment and location of subunits in bovine mitochondrial Complex I (Figure 2, Figure 3) [56•, 58•, 60•, 64•]. In the end resolution is just a number. If a biological question can be addressed with a medium resolution map then perhaps time can be better spent on other problems.

## Present limits and possibilities of single particle cryoEM

**Figure 4:**
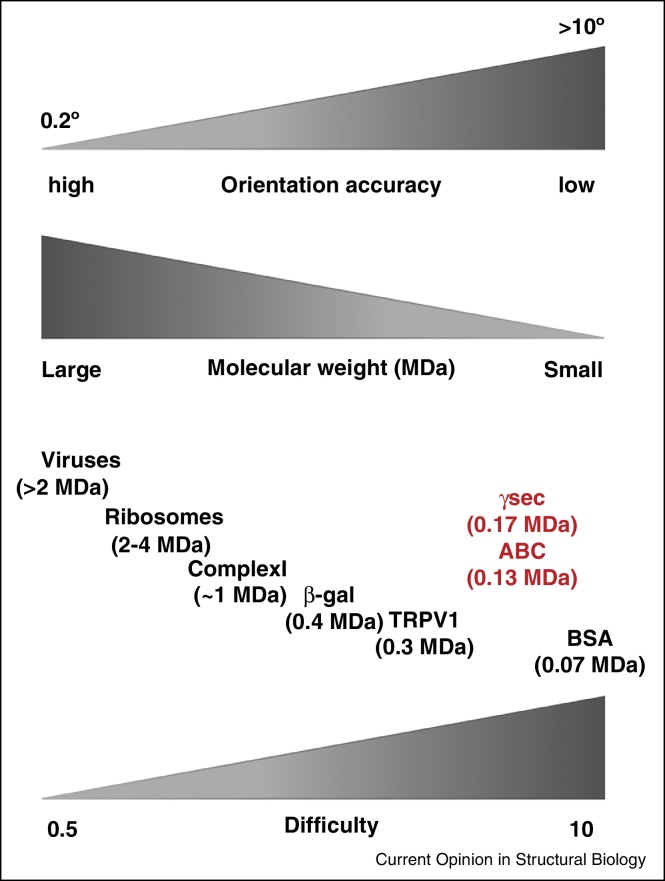
Structure determination of macromolecules by single particle electron cryomicroscopy: present limits and possibilities. With the present technology that includes microscopes with better vacuum, stable stage, coherent beam, direct electron detector, software and computing we can now achieve high-resolutions of macromolecules by single particle cryoEM. Although, the theory says that structure determination of macromolecules can be done with less particles and smaller proteins, currently there are limits to what is possible [16^••^]. As explained in the introduction of the main text, the quality of the map/reconstruction for a macromolecule depends on how accurately it can be oriented. Large symmetrical molecules such as viruses can be oriented accurately between 0.2 and 0.5 degrees and maps below 4 Å can now routinely be obtained with around 1000 particles (due to symmetry the number of asymmetric units averaged will be 60,000). Ribosomes with their bound RNA have higher contrast and are less sensitive to radiation damage. They have been one of the test specimens in the development of single particle EM. The resolution of ribosome maps has been gradually increasing and multiple reconstructions with many different factors now dominate the electron microscopy data bank (EMDB). Thus, in terms of difficulty high-resolution structures of large symmetrical molecules and high contrast objects such as ribosomes can be obtained with relative ease. As the size of the macromolecule becomes smaller, it is generally more difficult to obtain high-resolution structures and requires a lot more effort. Small protein molecules such as hemoglobin or bovine serum albumin (BSA) can be visualized in micrographs but presently cannot be oriented accurately. Recent structures of γ-secretase (170 kDa) and ABC transporter (130 kDa + 50 kDa Fab fragment) are highlighted in red to show the smallest asymmetric structures determined to sub-nanometer resolution by cryoEM at the moment [56•, 59•]. The number of particles required for a given protein to reach a resolution beyond 4 Å will depend on various factors but will largely be determined by its stability and heterogeneity and one could expect to average 10^4^–10^6^ asymmetric units to achieve a resolution that can resolve side chains for a wide range of molecules.

A question that is often asked is the lower limit in the size of the protein for which a structure can be obtained by single particle cryoEM. According to theory, structures of proteins as small as hemoglobin (∼64 kDa) can be obtained [16^••^]. While such small proteins can be visualized in micrographs, they cannot be oriented reliably with the present technology. Figure 4 tries to summarize the current possibilities with cryoEM for macromolecules in general. Large symmetric molecules such as viruses can be oriented with high accuracy and at present a resolution of 4 Å or above can be obtained by averaging only few thousand particles requiring less than half day of data collection. As the size of the molecule decreases the difficulty in obtaining such high-resolution structures increases and often require averaging of 10^4^–10^6^ of asymmetric units. Dynamic and structurally heterogeneous membrane proteins such as the rotary ATPase's may require much more particles to classify distinct conformations and may still only give lower resolution maps [60•, 64•].

The structure of the 130 kDa heterodimeric ATP-binding cassette (ABC) transporter from *T. thermophilus* is to date the smallest asymmetric protein structure that has been determined to below 10 Å by single particle cryoEM [59^•^]. This was possible by the addition of a 50 kDa Fab fragment, which binds to one of the monomer thus not only increasing the size of molecule but greatly aiding the orientation determination [59^•^]. Similarly, in the multi subunit complex of 170 kDa γ-secretase, the presence of a large ectodomain helps in alignment of the molecule [56^•^]. For small membrane proteins that may look like spherical blobs (in particular if the membrane part is covered with a large amount of detergent) it is possible that addition of Fab fragment or small domains may become a generic technique to obtain accurate orientation [79]. These examples represent what is currently possible with cryoEM and highlight that not only the size but also the shape matters for obtaining high-resolution maps by single particle EM.

## Conclusions and outlook

Recent progress in the field of cryoEM has been aptly called a ‘resolution revolution’ [80]. Some of the recent examples show that it is possible to reach beyond 3 Å [62•, 81, 82, 83]. While such resolutions are remarkable, it does involve averaging a large number of particles, in the range of 10^5^–10^6^ asymmetric units (Table 1). From theory it is clear that similar resolutions should be obtained with fewer particles [16^••^]. Analysis of the movie frames from direct electron detectors reveals that the first few frames are worse and have much less information than expected [24, 84]. This is one of the major reasons why the images are still not perfect and why the averaging of so many particles is still required. In current refinement strategies, this is overcome either by discarding those frames [23, 82] or by down-weighting them [24, 84]. Looking ahead, the next technical advances in cryoEM will come from two fronts. Firstly, understanding and curing the problems associated with the initial frames and secondly, the next generation of direct electron detectors with even higher DQE.

With respect to membrane proteins, won’t it be wonderful if it becomes possible to visualize and obtain structures of membrane proteins *in situ* in their native environment? For instance, ion channels at the synapse. The technique of electron cryotomography, which has been developed for a very long time, has yielded stunning images of whole cells and thin sections [85]. With the direct electron detectors and sub-tomogram averaging, it is now possible to obtain structures of macromolecules in their native environment, sometimes to sub-nanometer resolution [86, 87]. Recent analysis of the ATPase dimers in the inner mitochondrial membrane, membrane associated mitochondrial ribosomes and the nicotinic acetylcholine receptor with rapsyn on the post-synaptic membranes of the electric organ of *Torpedo* highlight such possibilities for membrane proteins [88, 89, 90]. Two recent advances namely the gold supported grids that minimize some beam-induced movement [91•, 92•] and the phase plate [93, 94] have great potential in acquiring better images by tomography and increased resolution by sub-tomogram averaging.

The future of cryoEM looks very promising and once a few technical problems are overcome, in the near future it may be possible to collect multiple data sets in a single day just like the present day data collection from crystals at synchrotrons.

## Conflict of interest

Nothing declared.

## References and recommended reading

Papers of particular interest, published within the period of review, have been highlighted as:

• of special interest
•• of outstanding interest

## Figures and Tables

**Table 1:** Compilation of membrane protein structures determined by single particle electron cryomicroscopy to 10 Å or below (till April 2015)

Protein	Source	Molecular mass (in MDa)	Medium	Detector	No. of particles^a^	No. of asymmetric units^a^	Resolution (Å)^b^	EMDB	Ref
Complex I	Native (bovine)	1	Cymal-7	Falcon II	25,492	25,492	∼5	2676	[58^•^]
V/A-ATPase	Native (*Thermus thermophilus*)	0.6	DDM	Film	46,105	46,105	9	5335	[47^•^]
	Native (*Manduca sexta*)^c^	0.9	C_12_E_10_	Falcon II	6714	6714	9.4	2781	[61^•^]
	Native (*S.cerevisiae*)	0.9	DDM	K2	50,030	50,030	6.9^d^	6284	[64^•^]
F_1_F_o_-ATPase	Native (*Polytomella sp.*)	1.6	DDM	Falcon II	37,238	74,476	∼7	2852	[60^•^]
Glutamate receptor^c^	Recombinant	0.4	DDM	Falcon II	21,360	42,720	7.6	2685	[57^•^]
Ryanodine receptor^c^	Native (rabbit skeletal muscle)	2.2	Nanodisc	Falcon II	25,000	100,000	6.1	2751	[38^•^]
			Nanodisc	TVIPS F816	94,354	377,416	8.5	2752	[38^•^]
			CHAPS/lipids	K2	46,400	185,600	4.8	6106	[39^•^]
			Tween-20	Falcon II	65,872	263,488	3.8	2807	[37^•^]
			DDM	CCD	28,036	112,144	**9.5**	1275	[45]
			CHAPS	Film	25,722	102,888	**10.2**	5014	[46]
TRPV1	Recombinant	0.3	Amphipol A8-35	K2	37,310	149,240	3.3^d^	5778	[55^••^]
TRPA1	Recombinant	0.7	PMAL-C8	K2	20,733	82,932	∼4^d^	6268	[63^•^]
γ-Secretase	Recombinant	0.17	Amphipol A8-35	K2	144,545	144,545	4.5	2677	[56^•^]
			Digitonin	K2	177,207	177,207	4.3	2974	[65]
Tmr AB+ AH5	Recombinant	0.18	DDM	K2	102,000	102,000	8.2	6085	[59^•^]
Tmr AB	Recombinant	0.135	DDM	TVIPS F816	36,000	36,000	10	6087	[59^•^]
Anthrax prepore toxin	Recombinant	0.44	NP-40	K2	60,455	423,185	2.9	6224	[62^•^]
Ribosome complexes^e^		2.6–4.3							
Sec61	Native (porcine)		Digitonin	Falcon II	80,019	80,019	3.35–3.9	2644, 46, 49, 50	[66]
	Native (*Canis sp*.)		Digitonin	TVIPS F416	162,655	162,655	**6.9**	2510	[50]
Ssh1	Native (yeast), Ssh1(R)		Digitonin	Film	183,000	183,000	**6.1**	1651	[48]
SecYEG	Native (*E. coli*)		Nanodisc	Film	85,664	85,664	**7.1**	1858	[49]
	SecYEG (R)								
SecYEβ	Native (*M. jannaschii*)		DDM	Film	37,000	37,000	**9**	5691	[53]
	SecYEβ (R)								
Sec61+OST+TRAP	Native (Triticum or *Canis sp.*)		Digitonin	TVIPS F416	15,705	15,705	**9.3**	2523	[52]
			Digitonin	Film	79,000	79,000	**8.7**	1528	[51]
YidC	Native (*E. coli*) YidC (R)		DDM	TVIPS F416	58,960	58,960	**8.0**	2705	[54]

a Number of particles denotes the number used in the final map and number of asymmetric units is the total number averaged after the application of symmetry.

b The criteria used to estimate the resolution is the Fourier shell correlation (FSC) either at FSC at 0.143 or 0.5. The numbers denoted in bold are reported at FSC 0.5.

c The structure of V-ATPase from *Manduca sexta*, glutamate receptor and the early structures of ryanodine receptor (taken on film or CCD) have an overall resolution of 10 Å or better. However, the membrane domain is of lower resolutions and TM helices are not clearly resolved.

d Multiple maps of V-type ATPase, TrpV1 and TrpA1 have been deposited and only the highest resolution is shown in the table. Three conformational states of V-ATPase have been resolved and they are deposited in EMDB codes 6284–86. The structures of TrpV1 with ligands are deposited in EMDB codes 5776–77 and please refer to Cao *et al.* 2013 for details of the structures [95^•^]. Similarly, the structures of TrpA1 with other ligands are deposited in EMDB codes 6267 and 6269.

e A collection of membrane proteins in complex with ribosomes are listed here. There are many more maps deposited in the database describing different states and the list here is only a small collection chosen based on the species and describing only the highest resolution map. The resolution shown is the overall resolution and in many of these maps, the membrane protein is of lower resolution, sometimes the TM-helices in the membrane domain is not clearly resolved. The translocon proteins SSh1, SecYEG (*E. coli*) and SecYE (*M. jannaschii*) and YidC are all recombinant (shown in brackets as ‘R’), while the ribosomes used in the study are native. Abbreviations used in the table: EMDB: electron microscopy data base; DDM: *n*-dodecyl-β-maltopyranoside; Cymal-7: 7-cyclohexyl-1-heptyl-β-maltoside; PMAL-C8: poly(maleic anhydride-alt-1-decene) substituted with 3-(dimethylamino) propylamine; C_12_E_10_: polyoxyethylene(10) dodecylether; NP-40: Nonidet P-40; OST: oligosaccharyl-transferase; TRPV1: transient receptor potential V1; TRPA1: known for its extensive ankyrin repeats at the amino terminal domain; TRAP: translocon associated protein complex.
